# A Gamification Framework for Cognitive Assessment and Cognitive Training: Qualitative Study

**DOI:** 10.2196/21900

**Published:** 2021-05-18

**Authors:** Ali Khaleghi, Zahra Aghaei, Mohammad Amin Mahdavi

**Affiliations:** 1 Department of Computer Engineering Imam Khomeini International University Qazvin Iran

**Keywords:** cognitive tasks, boredom, motivation, gamification, game elements, framework, process, gamification design, cognitive training, cognitive assessment

## Abstract

**Background:**

Cognitive tasks designed to measure or train cognition are often repetitive and presented in a monotonous manner, features that lead to participant boredom and disengagement. In this situation, participants do not put forth their best effort to do these tasks well. As a result, neuropsychologists cannot draw accurate conclusions about the data collected, and intervention effects are reduced. It is assumed that greater engagement and motivation will manifest as improved data quality. Gamification, the use of game elements in nongame settings, has been heralded as a potential mechanism for increasing participant engagement in cognitive tasks. Some studies have reported a positive effect of gamification on participant performance, although most studies have shown mixed results. One reason for these contrasting findings is that most studies have applied poor and heterogeneous design techniques to gamify cognitive tasks. Therefore, an appropriate gamification design framework is needed in these tasks.

**Objective:**

This study aimed to propose a framework to guide the design of gamification in cognitive tasks.

**Methods:**

We employed a design science research (DSR) approach to provide a framework for gamifying cognitive assessments and training by synthesizing current gamification design frameworks and gamification works in cognitive assessment and training, as well as incorporating field experiences. The prototypes of the framework were iteratively evaluated with 17 relevant experts.

**Results:**

We proposed a framework consisting of 7 phases: (1) preparation; (2) knowing users; (3) exploring existing tools for assessing or training a targeted cognitive context and determining the suitability of game-up and mapping techniques; (4) ideation; (5) prototyping using the Objects, Mechanics, Dynamics, Emotions (OMDE) design guideline; (6) development; and (7) disseminating and monitoring.

**Conclusions:**

We found that (1) an intermediate design framework is needed to gamify cognitive tasks, which means that game elements should be selected by considering current cognitive assessment or training context characteristics since game elements may impose an irrelevant cognitive load that, in turn, can jeopardize data quality; (2) in addition to developing a new gamified cognitive task from scratch, 2 gamification techniques are widely used (first, adding game elements to an existing cognitive task and second, mapping an existing game to a cognitive function or impairment to assess or train it); and (3) further research is required to investigate the interplay of cognitive processes and game mechanics.

## Introduction

### Background

Statistics show that the cognitive assessment and training market will achieve a growth rate of 32.39% from 2018 to 2022, and gamification will be one of the key vendors operating in this market [1]. These statistics confirm another estimation that shows 1 out of 5 people will be over 60 years old in the next 40 years [2]. Minor and major neurocognitive disorders, which have prevalences of approximately 10%-20% and 5%-7%, respectively, are global health issues due to the aging population; according to the American Psychiatric Association Diagnostic and Statistical Manual of Mental Disorders, edition 5, minor and major neurocognitive disorders encompass mild cognitive impairment and dementia, respectively [3]. In minor neurocognitive disorders, cognitive abilities decline, but changes are not severe enough to significantly affect individuals’ activities of daily living (ADLs); 35% of individuals with a minor neurocognitive disorder progress to a major neurocognitive disorder within a 3-year period [4]. In this stage, individuals lose independence in their ADLs and require care and support from others. It is not only aging that causes cognitive impairments; they may also exist since childhood (such as attention deficit hyperactivity disorder [ADHD] and autism spectrum disorder) or because of different factors like alcohol or drug abuse [5-7].

**Figure 1:**
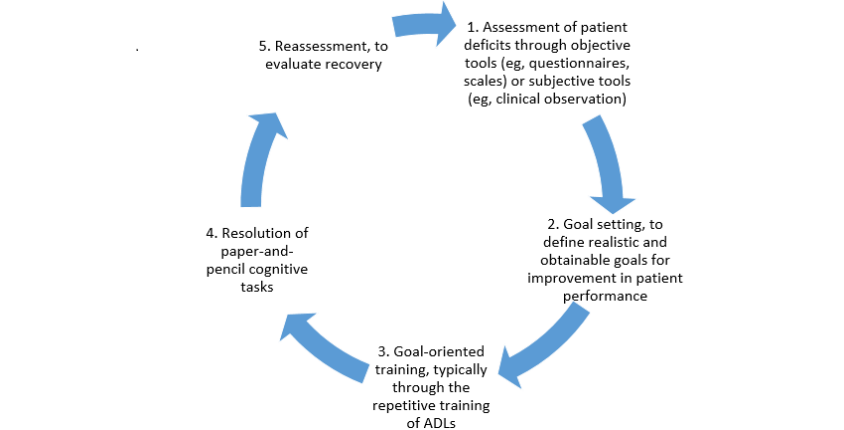
The process of traditional cognitive training programs (the figure was drawn based on descriptions provided by Vourvopoulos et al [8]). ADLs: activities of daily living.

Cognitive assessment and training play an essential role in preventing loss of autonomy and independence in ADLs [8]. Cognitive assessment is associated with evaluating individuals’ cognitive abilities (eg, working memory, attention, executive functions) [9]. Cognitive training refers to using cognitive tasks to maintain or improve a particular aspect of cognitive functioning [9]. There are cognitive tasks to assess or train cognitive functions. Cognitive tasks such as the Mini-Mental State Examination (MMSE) [10], Confusion Assessment Method (CAM) [11], and Montreal Cognitive Assessment (MoCA) [12] are widely used for cognitive assessment. There are also cognitive training companies that have developed cognitive training tasks such as Cogmed [13], Nintendo Brain Age [14], Lumosity [15], and Posit Science BrainHQ [16]. The prevailing approach in these companies for designing a training task is to convert lab-based and individualistic cognitive assessment tasks into a training task [17] (eg, Nintendo Brain Age includes several cognitive assessment tasks like the Stroop task [18]). Cognitive tasks are a vital tool for the assessment and training of cognitive impairments. However, participants often view them as monotonous and boring since they have a repetitive nature and are rigidly presented [8,9,19-23]. These features increase the frequency of insufficient efforts to perform these tasks, and consequently, the reliability of data collected decreases [9,19,23-26]. The typical process taken by cognitive specialists to perform a cognitive training program is shown in Figure 1.

**Figure 2:**
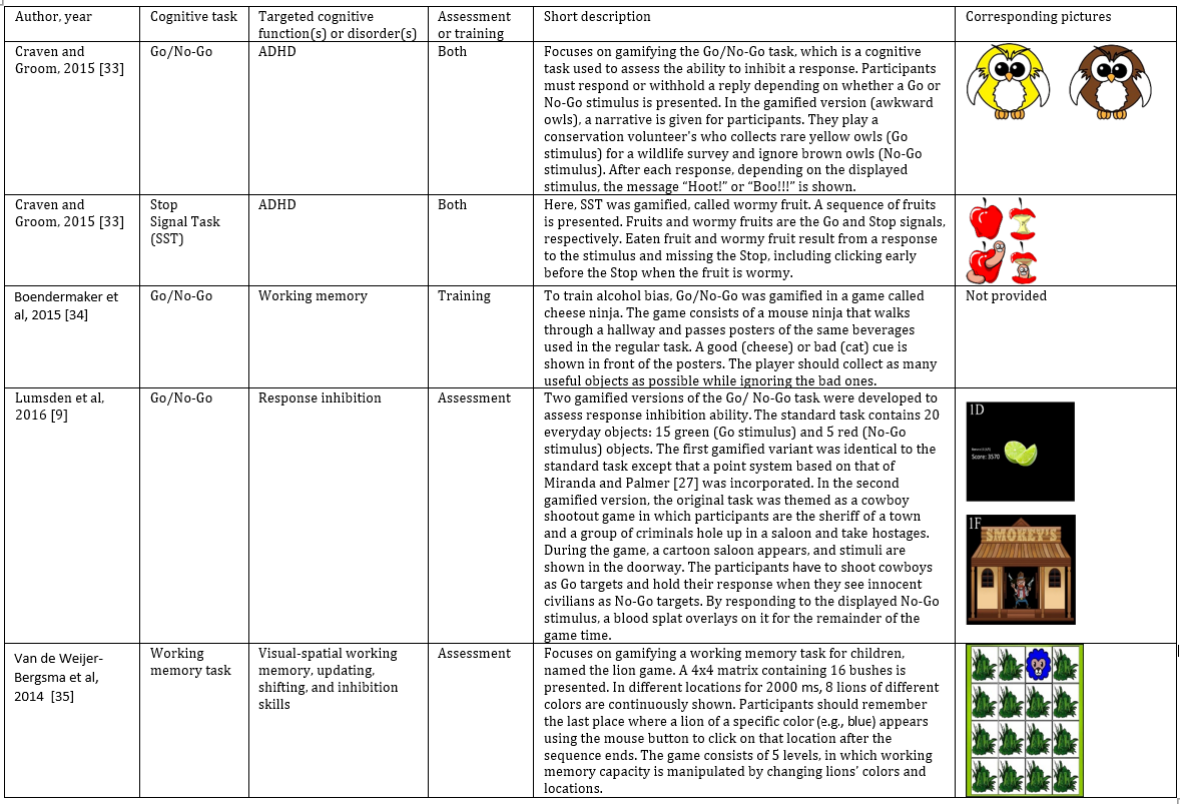
Examples of gamified cognitive tasks. A task was categorized as an assessment or training according to the self-prescription provided by the authors of the publication. The owl and apple pictures were used or adapted by [33] from openclipart [36] and 4vector [37] (online media repository of free graphics). ADHD: attention deficit hyperactivity disorder.

It is assumed that greater engagement and motivation will manifest as improved data quality in cognitive tasks [9,19-27]. Among the existing solutions, gamification, which is the process of adding game elements (eg, scoring system, leaderboard, badge) to nongame contexts (eg, education context, business context, cognitive tasks) [28], stands as one of the most influential and promising solutions to improve motivation in monotonous tasks [9]. A greater understanding of human motivation helps maintain users' encouragement to participate in cognitive tasks over time [17]. Motivation is multidimensional and falls on a continuum from intrinsic motivation to extrinsic motivation to amotivation (little to no motivation exists) [29]. Intrinsic motivation is regulated internally and refers to performing activities for their inherent satisfaction. Intrinsic motivation is required for long-term engagement and long-term changes. In contrast, extrinsic motivation (doing activities solely for their outcomes and rewards) is useful for short-term engagement and short-term changes and is also regulated externally [29,30]. Self-determination theory (SDT) [31] and Flow [32] theory are widely used to improve users’ participation and motivation. According to SDT [31], intrinsic motivation can be sustained by satisfying 3 psychological needs of relatedness (is experienced when individuals feel connected to others), autonomy (the need for freedom to make choices based on one’s volition during an activity), and competence (the need for challenge and feelings of self-efficacy). Flow refers to “the state in which people are so involved in an activity that nothing else seems to matter; the experience itself is so enjoyable that people will do it even at great cost, for the sheer sake of doing it” [32]. Gamification can combine intrinsic motivation with extrinsic motivation to raise motivation and engagement [30]. Game elements such as badges, points, game levels, a leaderboard, and avatars lead to extrinsic motivation and are useful for capturing early user motivation [17]. Based on SDT [31] and Flow [32] theory, gamification can also improve the intrinsic motivation of participants through game elements such as optimal challenges and positive feedback (these elements satisfy human needs of competence). Figure 2 presents some examples of gamified cognitive tasks from Craven and Groom [33], Boendermaker et al [34], Lumsden et al [9], and Van de Weijer-Bergsma et al [35].

### Challenges With Designing Gamification for Cognitive Assessment or Training

Despite the growing trend towards using gamification in cognitive tasks, its impacts on participant engagement and data quality are not stable. Studies have [20-22,27,38-40] stated that gamification has a positive effect on users’ engagement as well as data quality. However, other studies [19,24,26,34] have reported that no effects were observed on users’ performance by adding game elements, but they perceived the gamified task as funnier and more challenging than the nongamified version. In addition, other studies [19,27] showed that gamification worsened data quality but had positive effects on engagement. The gamification applied by Birk et al [23] not only did not have a positive impact on data quality and engagement but also worsened them [19]. These mixed findings are potentially due to 4 main reasons: (1) Most gamified cognitive tasks have been developed by cognitive psychologists, not professional gamification designers, and for scientists, the clinical effectiveness of a gamified task is important, with less focus on employing effective and creative gamification designs [41]; (2) a variety of gamification techniques have been applied to cognitive tasks [9,24,38]; (3) gamification techniques have been applied to different cognitive tasks [24]; and (4) the results obtained from gamified cognitive tasks are often preliminary and limited by small sample sizes. Also, the considered duration for evaluating the efficacy of gamified tasks is relatively short [9,23-25,42,43].

There are recommendations and design guidelines to integrate game elements into cognitive tasks (eg, [9,30]). However, to our knowledge, they did not propose a detailed and step-by-step framework that clearly shows what factors are essential to designing gamification in these tasks from early stages (eg, planning and preparation phases) to develop, evaluate, and disseminate gamified tasks, followed by monitoring the efficacy of such tasks in the long term. In general, several gamification frameworks have been developed by experts, not designed for cognitive tasks (eg, [44-53]). However, they suffer from 3 main limitations: (1) Most of them have been designed for enterprise and business contexts, with less focus on health contexts [54]; (2) cognitive tasks need to engage participants for the long term, but existing frameworks have not been designed for this purpose [54,55]; and (3) they have not specified how game elements should be added to a particular context. Incorporating game elements in cognitive tasks may jeopardize data quality by imposing an additional cognitive load to these tasks [56]. For example, Katz et al [25] gamified the N-Back task by including a real-time scoring system while completing the task. The gamified task, in comparison to the actual task, negatively impacted data quality. One possible explanation is that the game features imposed irrelevant cognitive demands by distracting the players’ attention.

### Objectives

Despite the papers that have shown mixed findings of using game elements in cognitive tasks, we assume that gamification can positively influence data quality and user engagement. Therefore, we are proposing a framework to guide the process of incorporating game elements in cognitive tasks by synthesizing (1) existing gamification design frameworks, (2) gamification efforts in cognitive assessment and training, and (3) field experiences.

## Methods

### Overview

**Figure 3:**
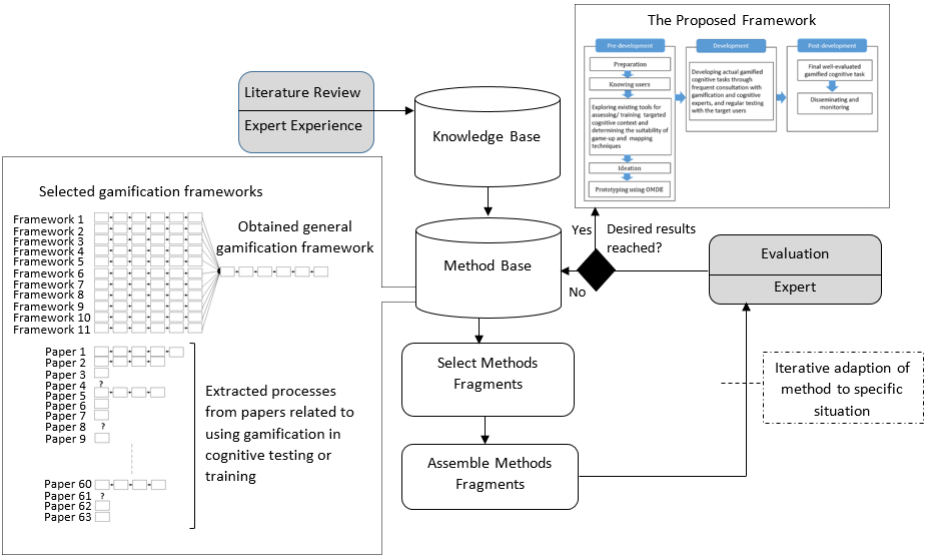
Situational method engineering approach followed for developing the framework (adapted from [53,59]).

We approached the research problem through the design science research (DSR) methodology [57]. Design science is an accepted research methodology in information systems. It emphasizes that research should be firmly grounded in existing knowledge and target the context in which the developed artifact must be used, to create scientifically sound artifacts (eg, theories, models, and methods) [58]. The 2 main steps for conducting DSR include (1) developing artifacts and (2) evaluation of the developed artifacts [53]. In our DSR approach, similar to Morschheuser et al [53], we employed an assembly-based situational method engineering methodology proposed by Brinkkemper [59]. Method engineering is an approach in information systems to develop new methods from recognized fragments of existing methods knowledge to propose a situational method tuned to the situation of the project at hand. According to method engineering, 3 phases are needed to develop a new method [59]: (1) building a method database, which includes all the resources required for the development of a new situational method; (2) constructing the situational method through assembling of the methods fragments from the method database; and (3) evaluation of the developed method. Figure 3 provides an overview of our assembly-based situational method engineering.

### Knowledge Base

#### Defining Sources of Evidence

We selected the following 3 resources to gather the knowledge required for proposing the intended framework: (1) studies that proposed a gamification design framework, since by synthesizing them, we could extract the general framework for the design of gamification; (2) projects that used gamification in cognitive assessment or training to extract key factors and considerations for gamifying these contexts; (3) the experiences of relevant experts to integrate the evidence from live environments.

#### Exploring Relevant Papers

We used systematic literature review strategies in the Google search engine, Google Scholar, PubMed, and Research Gate to use a wide variety of relevant papers. The search was started in May 2017 and lasted until the submission date. Two search strings were developed based on keywords, their synonyms, and related terms: (1) (“gamification” AND (“framework” OR “platform” OR “process” OR “method”)) and (2) (“gamification” OR “serious games” OR “game up” OR “video game”) AND ((“cognitive” AND (“training “OR “assessment”)) OR “Go /No-Go” OR “N-Back” OR “MMSE” OR “MoCA” OR “ADHD” OR “stop-signal task” OR “dyslexia”). Furthermore, search strategies such as checking the reference lists of included studies and cited reference searching were applied.

#### Inclusion Criteria for Selecting Gamification Frameworks

To select the most highly regarded gamification frameworks, the following 4 metrics were used: (1) framework was not focused on the parts or steps of the gamification design process, but covering the maximum number of steps; (2) framework was determined to be worthy in terms of efficacy by calculating the number of its citations; (3) framework was developed by gamification experts (we considered an individual an expert based on whether she or he published at least 10 scientific articles concerning gamification issues); (4) framework was developed using a robust methodology.

#### Inclusion Criteria for Selecting Gamification Projects in Cognitive Assessment and Training

First, we included projects that published reports about the impacts of game elements on data quality and user engagement in the form of a scientific paper, to obtain sufficient author-presented analytical expressions on how game elements should be incorporated into cognitive tasks. Second, similar to Lumsden et al [9], we did not select projects based on whether they included the word “gamification”; instead, we selected projects if our search strategies found them. We intentionally did not define gamification as defined by Deterding et al [28] (“the use of game design elements in non-gaming contexts”), since precisely defining the elements that make a game is challenging and limiting [9]. Therefore, we decided that a cognitive task was gamified if its purpose was to increase participants’ commitment and motivation. However, it used other game-inspired designs such as serious games, video games, games with a purpose, and game-like interventions. We erred on the side of caution to minimize the potential loss of relevant sources.

#### Expert Evaluation

From the early stages, the framework was screened and judged by a homogeneous group of 17 experts from relevant disciplines, including information technology, game, gamification, and cognitive psychology. The average years of experience are presented in Table 1.

**Table 1:** Experts’ background and average years of experience.

Background	Average experience (years)
Information technology (IT)	20
Game	4/5
Gamification	3
Cognitive psychology	11 years of academic experience and 6 years of clinical experience

After extracting each piece of evidence and then applying it to the under-development framework, the whole of the framework was visualized for expert evaluation. Then, the framework was refined based on the feedback collected.

### Method Base

#### Extracting the General Gamification Framework

After comparing the selected frameworks in terms of main characteristics, merits, and demerits, 2 general gamification design frameworks were elaborated. The first framework was based on analyses presented in Multimedia Appendix 1. The second framework was based on the Mechanics, Dynamics, Aesthetics (MDA) process, a formal framework for designing and analyzing games [60], and 2 adapted versions of MDA presented in [44,52], now known as Objects, Mechanics, Dynamics, Emotions (OMDE). The phases and activities of these frameworks will be described in detail in the subsequent sections while describing the proposed framework.

#### Customizing the General Gamification Frameworks for Cognitive Assessment and Training

An exhaustive number (n=63) of empirical project reports or theoretical works that applied gamification into cognitive tasks were gathered to customize the obtained general frameworks for cognitive assessment and training contexts. We tried to extract an abstract process for each paper by observing the papers’ different sections. As abstracted in Figure 3, most articles did not use a specific or formal framework for gamifying cognitive tasks. Therefore, either we could not extract a process, or the process obtained consisted of only one chunk. Finally, the isolated processes, fragments, and general gamification frameworks were converged based on their commonalities and unique features to assemble the intended framework. 

## Results

### Search Results

#### The Explored Gamification Design Frameworks

We identified a total of 35 gamification design frameworks (these frameworks are listed in Multimedia Appendix 1). Of these, 11 frameworks were selected for more in-depth analysis (these frameworks are highlighted in bold in Multimedia Appendix 1).

#### The Explored Gamification Projects in Cognitive Assessment and Training

A total of 63 gamification projects in cognitive assessment or training was selected. Of these, 41 (41/63, 64%) were empirical project reports; 2 (2/63, 3%) used empirical and theoretical methods, and 20 (20/63, 32%) were theoretical. As for the purpose of the papers, 22 (22/63, 35%) were for assessment, 32 (32/63, 51%) for training, and 9 (9/63, 14%) for both assessment and training. In addition, 19 (19/63, 38%) were for children aged 3-14 years; 16 (16/63, 34%) for adolescents, youth, and adults aged 15-55 years; and 13 (13/63, 28%) for older adults aged 56-94 years. For more details and raw data about the works included, such as their targeted cognitive functions or impairments, please see both tables in Multimedia Appendix 2.

### The Proposed Framework

#### Overview

**Figure 4:**
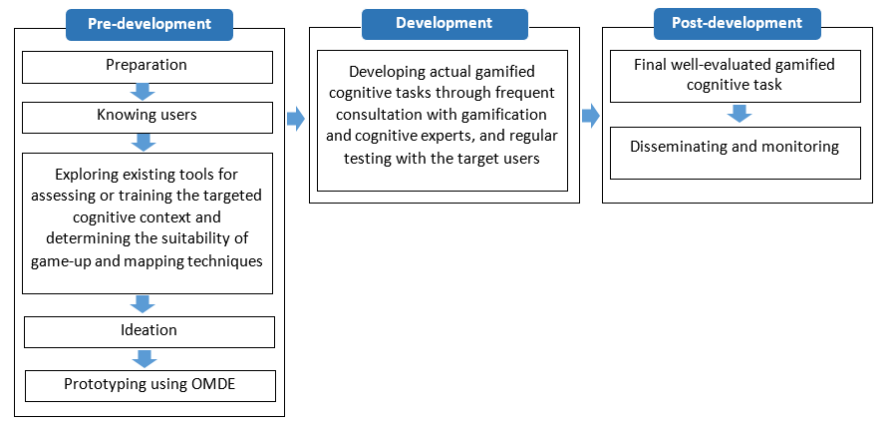
The proposed framework. OMDE: Objects, Mechanics, Dynamics, Emotions.

To address the need for a framework that more effectively integrates game elements into cognitive assessment and training, we introduce a framework consisting of 7 phases: (1) preparation; (2) knowing users; (3) exploring existing tools for assessing or training the targeted cognitive context and determining the suitability of game-up and mapping techniques; (4) ideation; (5) prototyping using OMDE; (6) development; and (7) disseminating and monitoring. These phases are grouped into 3 overarching categories: predevelopment, development, and postdevelopment (Figure 4). Although the framework phases are presented sequentially, they are not necessarily to be conducted linearly since different ideas and directions may be explored while integrating gamification into targeted cognitive tasks. Therefore, projects are encouraged to loop back through the phases continuously [61,62].

The framework aims to facilitate creating more effective gamified cognitive tasks by using an interdisciplinary team of gamification designers, cognitive experts, and target users. Only a truly multidisciplinary team has the knowledge and expertise to address the complex factors involved in the design of gamification into cognitive tasks [61,63,64]. Gamification designers are not familiar enough with the process and execution of targeted cognitive tasks. As a result, they may incorporate game elements inappropriately [19,23-25,33,65]. Therefore, gamification designer and cognitive expert involvement is needed throughout the design process, and target users should be involved throughout phases 2, 4, 5, and 6. The involvement of target users in these phases places their needs and motivations at the center of attention. The first 3 framework phases are primarily about information gathering to develop a more well-accepted and scientific gamified task in later stages. Steps 4, 5, and 6 (ideation, prototyping using OMDE, and development, respectively) follow 2 main objectives: (1) generating gamification design ideas around targeted cognitive tasks (stages 4 and 5) and (2) developing actual gamified cognitive tasks through frequent consultation with gamification and cognitive experts and regular testing with target users (stage 6). Finally, once the efficacy of a gamified cognitive task has been demonstrated in phase 6, the task is disseminated to its target audience and then monitored periodically to maintain its effectiveness over the long term (stage 7).

#### Phase 1: Preparation

The primary purpose of the preparation stage is to have a good understanding of a gamification project’s objectives [53,66]. Defining objectives has been recommended in most reviewed gamification frameworks (9 of 11) and will support a later stage to figure out if the desired goals have been achieved [66]. The interdisciplinary team should list all potential objectives and then rank and justify the list in terms of importance since trade-offs of less important goals for more important ones might be needed [49,53]. Finally, as the team goes through gamification design and development, it can go back to the list to focus on what is really important [49]. Therefore, the defined objectives should be achievable, specific, relevant, measurable, and time-bound [66]. For instance, an initially broad goal of “increasing participants’ motivation to complete their cognitive training exercises” may be refined to “conduct 10 minutes of training each day.”

According to frameworks such as [50,53,66], gamification’s suitability as a possible way to intervene should be examined before starting any gamification design process. It can be carried out by detecting the problem that gamification should solve by gathering and analyzing quantitative and qualitative information. After determining the problem, the root reason that caused the problem must be motivational. Otherwise, gamification is not suitable [66]. The root reason can be identified by the “Five Whys” technique that determines the root cause of a problem by repeating the question “Why?” [67].

It is also essential to identify the standard project requirements and constraints such as scope (time, personnel, budget) and legal and ethical constraints since they can affect a gamification project’s success [50,53].

#### Phase 2: Knowing Users

During this stage, the interdisciplinary team must select one or more of a variety of methods to collect information about target users’ motivations and needs (eg, interviews, observation of target users’ behaviors, surveys, focus groups, questionnaires) [50,53]. After collecting and analyzing users’ data, users with similar characteristics should be segmented into groups to create user personas. The segmentation helps the team choose a more acceptable design to gamify targeted cognitive tasks.

Typically, gamification through motivational affordances enriches information systems [53]. Therefore, it is essential to conduct this phase (9 of 11 frameworks have had one step for understanding users). People are motivated by different motivational affordances based on characteristics such as their age, gender, and culture. The Octalysis gamification framework is widely used to segment users based on their motivations [68]. Octalysis was developed by Chou [68] as an octagon with 8 core drivers of individuals on each side: (1) epic meaning and calling, (2) development and accomplishment, (3) empowerment of creativity and feedback, (4) ownership and possession, (5) social influence and relatedness, (6) scarcity and impatience, (7) unpredictability and curiosity and, (8) loss and avoidance. The game strategies or elements that are associated with each driver have been grouped next to it.

**Figure 5:**
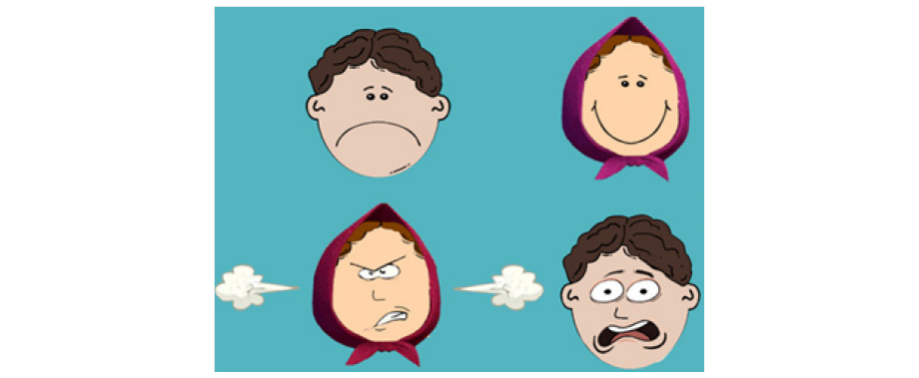
Screenshot of the game “Smile 1,” which was developed to help autistic children recognize different emotions [7].

In cognitive contexts, in addition to considering the users’ motivations, their needs should be identified since they may suffer from mild to severe cognitive dysfunction, which may sometimes be accompanied by physical disabilities. Afrasiabi Navan and Khaleghi [7] developed the game “Smile 1” to help Iranian autistic children recognize emotional states such as happiness, sadness, anger, and fear in the cartoon faces of girls and boys that appear in the game. The girls have a scarf (Figure 5) since these children only identify women and girls who wear a scarf (in Iranian culture, girls and women wear a scarf). To identify users’ needs, most gamified assessments and training have tried to implement a gamified experience that allows users natural and straightforward interactions, often using touch-based technologies such as smartphones and tablets (22 of 63). For more information about designing a user-friendly interface for people facing cognitive and physical disabilities, please see [2,69-73].

#### Phase 3: Exploring Existing Tools for Assessing or Training the Targeted Cognitive Context and Determining the Suitability of Game-Up and Mapping Techniques

At this stage, the interdisciplinary team should thoroughly acquaint itself with existing tools for assessing or training the targeted cognitive functions or impairments through methods such as consulting with cognitive experts and gathering quantitative and qualitative information. This work helps the team incorporate game elements into these tools without changing their process and execution and find integration points for adding game elements [74]. Describing the tools at a granular level is required. Otherwise, it is not guaranteed that the next framework steps will lead to the desired outcomes [53,74]. Three main tools that can be explored for cognitive assessment and training purposes are standard computerized or not computerized cognitive tasks, existing cognitive games, and existing video games.

For standard computerized or not computerized cognitive tasks, there may be more than one cognitive task designed to assess or train a cognitive function (eg, continuous performance test, Go/No-Go test, stop signal task for assessing attention, inhibitory, and motor skills). Selecting appropriate cognitive tasks is very important. Some tasks may have better performance than others [24,75]. Valladares-Rodriguez et al [75] chose the California Learning Verbal Test II to assess episodic memory. The task, in comparison to other tasks such as the Children’s Memory Scale, Rey Auditory Verbal Learning Test, and Wechsler Memory Scale, has a large number of variables and produces more qualitative information. Computer versions have now been made for many standard cognitive tasks, which are cheaper, more repeatable, and easier to administer and distribute [76]. Many of these tasks can be found in [77-79].

Regarding existing cognitive games, many games have been developed based on standard cognitive tasks [80]. It is beneficial to find these games since they can be reused for current cognitive assessment and training purposes, or the initial inspiration for gamifying current tasks can be obtained by reviewing the style of these games for integrating game elements into a cognitive task [25,33,43,75]. Brain games can be found from platforms such as Cogmed [13], Nintendo Brain Age [14], Lumosity [15], and Posit Science BrainHQ [16].

Regarding existing video games, it has also been demonstrated that classical video games of different genres that have not been inherently designed to assess or train cognitive functions can be reused as a standard cognitive task. Video game challenges come in various forms, and players have to use their underlying neural systems and cognitive abilities to win these games [81-90]. Each cognitive function is typically characterized by a set of parameters estimated from a gameplay to reuse for assessing and training. In other words, the team must identify which cognitive skills are central to each gameplay [91]. For winning games like Tetris and Candy Crush, mental rotation and spatial reasoning skills are required [91]. Card games like Solitaire and FreeCell have a reasonable correlation with classical measurements of executive functions and planning abilities [92,93]. The team can explore existing games from platforms such as the App Store and Google Play. They rank games based on their rate of downloads and players’ comments, which help select games according to the target user’s preferences. According to Green and Bavelier [83], Doherty et al [91], and expert experiences, it is unnecessary to find appropriate games through earlier methods for categorizing games such as genre-based methods since they are no longer effective. Games that have never overlapped in terms of content and mechanics now have many points of overlap [83].

After collecting the tools, the interdisciplinary team should determine whether game-up and mapping techniques can be used instead of designing a new gamified cognitive task from scratch. Game-up refers to adding game elements such as colors, animations, sound effects, and a backstory into standard cognitive tasks without changing their fundamental properties such as stimuli, design, and procedure [5,26,94] (21 of 63 studies used the game-up technique). The developed gamified cognitive tasks based on game-up are often presented in the form of a battery of mini-games. Each mini-game focuses on a specific cognitive function (eg, [64,76,95]). For example, Zeng et al [76] gamified a computerized cognitive test battery to detect impairments in 5 cognitive functions involved in developing a major neurocognitive disorder. For each test, some mini-games were designed in the context of ADLs such as cooking, cleaning, and shopping. The main feature of game-up is simplicity in terms of its mechanics and design [7,65,96,97]. This feature is useful for individuals who suffer from cognitive impairments like children with learning disabilities and ADHD who have weak working memory capacity [65,96]. Therefore, gamified cognitive tasks for these children should be broken into short and discrete tasks [65,96]. Mapping refers to reusing an existing game (cognitive and classical games) as a cognitive task and can save considerable time and effort that have been applied in the design of explored games [98,99]. The mapping technique was used by 21 of the 63 studies (eg, [72,92,93,100-104]). Explored games should be adjusted appropriately since they usually do not provide cognitive psychologists with sufficient quantitative data about the participant’s performance and progression on one hand. On the other hand, the used game elements and storylines may not align with participants' preferences [103,105,106]. Moreover, they may impose an additional cognitive load. Therefore, the exact cognitive demands of selected games should be identified by analyzing their structural characteristics [83]. Each game's structural characteristics should be examined individually since different games, even those that fall into one category such as action or first-shooting person, may require greatly different cognitive demands [81,85,107,108]. In mapping, it is also possible to mash up various games for cognitive assessment and training purposes [81]. For example, dyslexia is associated with a variety of underlying deficits in phonological, auditory, motor, memory, and visual attentional processes. According to previous findings showing the core deficit in dyslexia is related to attentional problems, Franceschini et al [81] explored 10 action games to train dyslexic children. Action games can enhance a wide variety of visual attentional abilities, such as segmenting items both in time and across space.

#### Phase 4: Ideation

The interdisciplinary team is involved in a highly iterative design process through the next 3 phases (ideation, prototyping using OMDE, and development). Iterative processes enable the team to obtain more creative and effective gamification designs. Of the 11 frameworks, 5 have one or more steps that should be iterated until the desired designs and outcomes are reached (ie, [46,48-50,53]). The steps that are often performed iteratively are ideation, prototyping, and development [53].

At this stage, the team combines the analyses and materials obtained in new ways to produce apt and innovative ideas to engage target users. It is necessary to involve a cross-functional group of people from cognitive experts, gamification designers, and target users to start this stage [61,62]. This work helps the interdisciplinary team to collect a greater number of more varied and creative ideas [61,62]. The participants should be encouraged to use different ways to be creative [61,62]. Brainstorming, co-creation workshops, and mind mapping are some methods [53,61,62]. The important question at this stage is how to help participants find the ideas. One solution is to explore existing games, gamification designs, and examples that may be a perfect fit for the current project [62]. For instance, in the game “Whack a Mole,” moles hide quickly, and the player trying to hit them with a hammer has to be faster. Various types of moles exist in different game versions, such as ninja, pirate, samurai, and batman moles [109]. Based on studies reusing the Whack a Mole to measure attention, inhibitory control, and executive functions [72,102,103], one idea is to use ninja and samurai moles as metaphors for Go and No/Go stimuli, respectively. As a result, a gamified cognitive task that mimics the Go/No-Go design can be created using the Whack-a-Mole style. Exploring many games and gamification examples and then mashing them up to fit the current problem is another right approach for generating ideas to gamify the current task [62].

After preparing ideas, similar ideas should be clustered using affinity diagrams; then, the clusters should be prioritized using methods such as dot voting. This work helps the team to focus on important ideas in the next 2 phases [50,53].

#### Phase 5: Prototyping Using OMDE

After collecting the right ideas, the interdisciplinary team needs to start prototyping. Prototyping is the stage in which the team implements the ideas into tangible forms to see how they actually work. In this stage, low-fidelity (ie, “quick and dirty”) prototypes are developed rapidly to gather feedback from relevant experts and target users early and often [61]. Prototyping saves time and resources by helping the team to identify refinements required before solidifying a design [61,62].

During each iteration of the prototyping, the team can use the OMDE design guideline to (1) check the motivational characteristics of prototypes (such as fun, flow, engagement, positive emotions) and (2) validate prototypes from cognitive psychology aspects. OMDE divides the components of a gamified cognitive task into 4 categories: objects, mechanics, dynamics, and emotions. Objects are a gamified cognitive task's assets, such as visual assets, images, audios, videos, and animations [60]. Mechanics refer to a gamified task's components at the level of game rules, algorithms, and data representation [60]. Dynamics are run-time users’ behaviors that emerge as users partake in the gamified task such as competition and cooperation [60]. Emotions refer to whatever emotions users experience while interacting with the gamified task [44]. Participants may experience different emotions such as fear, happiness, anger, sadness, and pride while interacting with the gamified task [44]. Dynamics and emotions emerge from the selected objects and mechanics [44,52,60]. For instance, a leaderboard mechanic leads to dynamics such as competition and comparison and emotions such as fear and happiness. Some participants may be afraid of being judged by others, and the use of the leaderboard may demotivate them from continuing the gamified task. Or, many participants may enjoy these dynamics, and the leaderboard can motivate them. Therefore, displaying participants' statuses in the leaderboard must be an optional feature in a gamified task.

Good dynamics and emotions are vital to ensuring a strong user commitment to participation [44,52,60]. To check gamified tasks’ motivational features with OMDE, the interdisciplinary team must first define the desired dynamics and emotional responses that the designed task should evoke among users. Then, in each iteration, the team must list what dynamics and emotions emerge from the gamified task in practice and then compare the responses with the desired ones to determine if the desired responses have been reached. The team cannot accurately predict what dynamics and emotions will emerge from a gamified task. Therefore, it is necessary to use OMDE iteratively [44,52].

In gamified cognitive tasks, it is also essential to validate the components of OMDE from cognitive aspects because they may impose an additional cognitive load. Objects and mechanics can cause difficulty in categorizing cognitive tasks’ stimuli for participants or can evoke emotions such as anxiety and stress that may distract participants’ attention from completing gamified tasks [19,23-25,33,65]. In this circumstance, participant errors increase, and the reliability of the data obtained decreases. For instance, in the study by Birk et al [23], the gamified Go/No-Go task decreased users’ performance. In the standard task, a sequence of stimuli is presented for 500 ms. Participants should respond to circles but not to squares. In the gamified version, participants should shoot blond zombies (Go stimulus) but not yellow hat moles (No/ Go stimulus). In the standard task, a circle is very different from a square. In contrast, in the gamified task, the colors of yellow hat moles and blond zombies are close to each other and can cause difficulty while participants are trying to identify the gamified task’s stimuli. In order to gamify the Go/No-Go task, Lumsden et al [19] suggested that red and green colors be used instead of cartoon characters because participants are more familiar with colors. The components of OMDE can be validated by discussions with cognitive experts and answering questions such as: (1) Is it possible to gamify the cognitive tasks’ stimuli? If yes, how can we do so? (2) Does the team have the freedom to choose objects for gamifying cognitive tasks, or should they be selected among those listed in a specified set or ones that participants are more familiar with, such as everyday objects? (3) What degree of structural similarities (such as shape, size, and color) between objects and mechanics should be adjusted? (4) Is it possible to gamify the surrounding environment of the selected cognitive tasks’ stimuli? If yes, how should the degree of separation between cognitive and gamified sections be adjusted? (5) Does the designed gamified cognitive task lead to negative emotions like anxiety and stress?

#### Phase 6: Development

**Figure 6:**
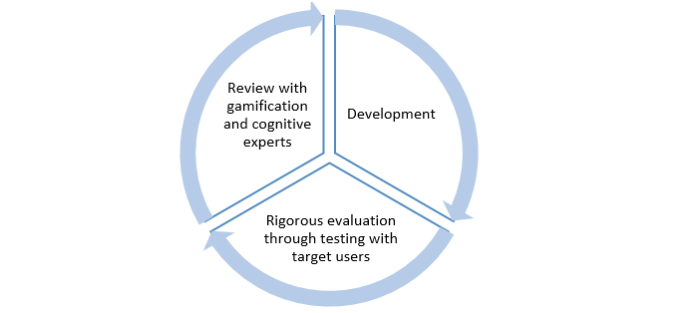
Overall structure of the development phase.

During this stage, actual gamified cognitive tasks are developed through frequent consultation with relevant experts (gamification and cognitive experts) and regular testing with target users (Figure 6). Based on the examined gamification efforts in cognitive tasks, to test the efficacy of gamified tasks, rigorous evaluations are required in terms of user engagement and data quality (eg, [9,19-21,23-27,34,38-40]).

Two methods are widely used to evaluate how gamified cognitive tasks influence users’ engagement and motivation [24,27,38,39]: (1) subjective measures of engagement, in which the motivation level of a gamified task is measured through self-report questionnaires based on SDT [31] and Flow [32] theory (eg, Flow State Scale [110]) and (2) objective measures of engagement such as the number of times participants used a gamified task or the number of optional cognitive assessment or training sessions performed by participants, methods that might be more preferred by the interdisciplinary team [24,38,39,63,111]. A combination of both methods is often used to measure gamified tasks’ motivation levels [24].

To assess how gamified cognitive tasks impact the quality of data and to indicate the maturity of these tasks to be used as a valid clinical tool, they must be evaluated for 2 essential properties [43]: (1) reliability, which refers to the extent to which a task's results are consistent and repeatable, and there are 4 types of reliability (test-retest reliability, parallel forms reliability, internal consistency reliability, and interrater reliability) and (2) validity, which refers to how well a task measures what it claims and includes criterion validity, content validity, construct validity, face validity, external validity, and ecological validity.

In cognitive training, it may also be necessary to measure to what extent gamified tasks can impact transfer effects [38,41,83]. The term “transfer” is frequently used in clinical practice and refers to the extent to which considered cognitive training tasks can improve untrained cognitive abilities. New tasks and situations are included to measure transfer effects. Transfer effects are divided into near and far transfer effects. Cognitive training has near transfer effects if it improves cognitive skills that are highly similar to trained cognitive skills. Far transfer effects refer to improvements in cognitive skills that are less similar to trained skills.

There are 2 other essential factors for conducting rigorous evolutions: (1) selecting sufficient sample sizes and (2) selecting an appropriate duration for evaluation. Most gamification efforts in cognitive contexts have used small sample sizes to evaluate the efficacy of gamified cognitive tasks [9]. Also, little consideration has been given to using statistical analyses such as power analysis for a sample size calculation [9,112]. For more information about how to calculate sufficient sample sizes, please see [113,114]. Insufficient sample sizes limit the reliability and generalizability of the results [9,115,116]. Moreover, only a few studies have used randomized controlled trials (RCTs) to evaluate gamified cognitive tasks [30]. In clinical research, RCTs are considered the most robust study design for evaluating the effectiveness of a new tool due to the ability of RCTs to minimize several forms of bias [61]. RCTs randomly assign participants to an experimental group and a control group. The use of an RCT design comparing gamified (experimental group) and nongamified (control group) versions of the same cognitive task has been highly recommended to evaluate the potential efficacy of gamified tasks [9,30]. Regarding selecting the appropriate duration for evaluation, participants are not involved in the gamified task over the long term but instead participate for a short time. In turn, it remains unclear after how long participants feel boredom and how the quality of data will alter in these circumstances [19,25]. Moreover, a short duration can cause errors due to participants' unfamiliarity with the gamified task. In this regard, using short tutorials and warm-up sessions before actual evaluation sessions has been recommended [65,76].

#### Phase 7: Disseminating and Monitoring

Once the gamified cognitive task's efficacy has been demonstrated in the previous step, the task is finally disseminated to its target audience. There have long been calls for disseminating and sharing well-evaluated digital health interventions due to the abundance of low-quality interventions currently available to the public [17,61]. Disseminating gamified tasks provides access to the broader population that may benefit the most from these tasks and helps the industry invest in these interventions more quickly [61,73]. Disseminating can occur via the App Store or Google Play. Also, industry partnerships can support a more effective and sustainable dissemination of gamified cognitive tasks [61,117]. It is also highly recommended that projects disseminate their findings, experiences, and methods for developing gamified cognitive tasks to scientific journals, conferences, researchers, and digital mental health intervention developers. It can advance future gamified tasks and improve their effectiveness [61,117]. Disseminating can also include open sharing of gamified cognitive task codes via GitHub [118] or allowing free access to a mobile health platform such as Mobile Sensor Data-to-Knowledge (MD2K) [119].

For disseminating gamified tasks, 2 other important factors should be considered by the team. First, according to expert experiences and [91] in collaboration with cognitive experts, appropriate guidelines and prescriptions should be prepared for using gamified cognitive tasks by clinics and target users (eg, determining the minimum effort and time that target users should spend to improve their cognitive skills). These instructions help mental experts and target users to use gamified tasks effectively. Second, ethical concerns are another primary concern that should be considered in this stage since performance variables are embedded in gamified tasks to track improvements in users’ cognitive functions. Therefore, gathering target users' information should be undertaken with their explicit consent [23,47,50,53,73].

After disseminating, gamified tasks’ performance should also be accompanied by postlaunch monitoring and evaluation, in which usage and applicability of the gamified task through methods introduced in the previous phases are observed in regular intervals to compile a list of possible improvements [47,53]. For instance, different levels of cognitive impairments may exist among users, and after a cognitive training program through the gamified task, users' cognitive status may improve. Therefore, according to the users’ new levels, some changes may need to be applied in the gamified task [69,73]. Also, gamified tasks’ motivation levels should be continuously monitored to maintain intrinsic motivation for the long term. The initial effects of game elements can diminish over time [47,53].

## Discussion

### Overview

This paper proposes a 7-step framework to guide the design, development, and evaluation of gamified cognitive tasks designed to assess or train cognition. Within these steps, there are a series of key recommendations on how each step should be operationalized. Along with the framework, the article presents the OMDE guideline at stage 5 of the framework (prototyping using OMDE), which contains vital recommendations for advancing the understanding of design complexities when applying gamification in cognitive tasks.

The prototypes of the framework were designed and evaluated extensively by evidence from 3 sources: (1) existing gamification design frameworks, (2) project reports of applying game elements into cognitive assessment and training, and (3) expert experiences. To our knowledge, this is the first study of its kind that has converged these sources to propose a unified model to design gamification in cognitive tasks. The significant point about gamification efforts in cognitive tasks is that they do not use a specific design process to incorporate game elements. We used an alternative solution, such that by observing each work, we tried to extract clues or pieces of information to propose an abstract process for each of them, if possible. This work helped us to identify critical factors and considerations for gamifying cognitive tasks.

### Comparison of the Proposed Framework With General Gamification Frameworks

Like general gamification frameworks, the proposed framework follows a user-centered design to improve users’ participation but has many added features that make it appropriate to gamify cognitive tasks. One main finding of this study that gamification designers need to be aware of is that an intermediate design is required for gamifying cognitive tasks, meaning that game elements cannot be selected without considering the targeted cognitive context characteristics. Otherwise, an irrelevant cognitive load may jeopardize data quality. Therefore, collaboration between both gamification and mental experts is required to examine the interplay of game elements and cognitive processes [9]. The intermediate design is a crucial feature that differentiates the gamification design framework required for cognitive assessment and training from other contexts. Based on examined gamification efforts in cognitive tasks, we concluded that 2 techniques have been widely used to gamify cognitive tasks besides designing a new gamified task from scratch: (1) gaming-up an existing cognitive task and (2) mapping an existing game (cognitive and classical games) to a cognitive function or impairment to assess or train it. The required details to use game-up and mapping techniques were provided in step 3 of the framework as far as possible.

### Limitations

The main limitation was that only a few studies have discussed how game elements impact participants when interacting with the gamified task and how they should be utilized in cognitive tasks to positively influence data quality and user engagement. Gathering evidence from the mentioned sources only gave an initial evaluation for the proposed framework because the number of experts and relevant studies was limited. Hence, a more robust evaluation is necessary. For this purpose, we listed most of the recognized experts in the area to evaluate and refine the framework in subsequent studies.

### Possible Future Studies

By further developing the framework exploited in this work and utilizing machine learning and deep learning algorithms, it is possible to create a recommender system that can suggest the most appropriate game elements according to characteristics of the targeted cognitive context and users' preferences or requirements. In our work, only existing games that have been developed or examined in scientific papers were investigated. It is also possible to analyze a great number of current games, from brain games to classical games, for further development of the framework. Due to the time constraints, establishing the feasibility of proposing different gamification design frameworks for cognitive functions that inherently share similar cognitive processes like processing speed (Gs) and working memory [20] was not provided.

### Conclusions

While more work is needed to further refine and evaluate the framework, we believe our framework has great potential to be used as a foundation for developing effective gamified cognitive tasks. Furthermore, ideas presented in the paper can be further developed and researched by many other researchers and practitioners.

## Appendix

Multimedia Appendix 1 Explored gamification design frameworks.

Multimedia Appendix 2 Included papers applied gamification in cognitive training/ testing.

## Abbreviations

ADHD: attention deficit hyperactivity disorder
ADLs: activities of daily living
CAM: Confusion Assessment Method
DSR: Design Science Research
MD2K: Mobile Sensor Data-To-Knowledge
MDA: Mechanics, Dynamics and Aesthetics
MMSE: Mini-Mental State Examination
MoCA: Montreal Cognitive Assessment
OMDE: Objects, Mechanics, Dynamics, Emotions
RCT: randomized controlled trial
SDT: self-determination theory
