# Supplementary material for: A Gamification Framework for Cognitive Assessment and Cognitive Training: Qualitative Study
Source: JMIR Serious Games. 2021 May 18;9(2):e21900. doi: 10.2196/21900 (PMC8170558; doi:10.2196/21900)
Supplement: Multimedia Appendix 1 [file games_v9i2e21900_app1.doc]

**Multimedia Appendix 1**

**Supplementary Table 1. The list of all explored gamification design frameworks (the bolded frameworks were those that selected for deeper analysis)**

| **Author, year** | **Full title** |
| --- | --- |
| **Hunicke, LeBlanc and Zubek, 2004** [1] | MDA: A formal approach to game design and game research |
| Charles and Black, 2004 [2] | Dynamic player modeling: A framework for player-centered digital games |
| Dignan, 2011 [3] | Game frame: Using games as a strategy for success |
| Radoff, 2011 [4] | Game On: Energize Your Business with Social Media Games |
| Kapp, 2012 [5] | The gamification of learning and instruction: game-based methods and strategies for training and education |
| **Aparicio, Vela, Sánchez and Montes, 2012** [6] | Analysis and application of gamification |
| **Werbach and Hunter, 2012** [7] | For The Win: How Game Thinking Can Revolutionize Your Business |
| Versteeg, 2013 [8] | Ethics & Gamification design a moral framework for taking responsibility |
| Marczewski, 2013 [9] | Gamification: a simple introduction |
| Merino de Paz, 2013 [10] | Gamification: A tool to improve sustainability efforts |
| Robinson and Bellotti, 2013 [11] | A preliminary taxonomy of gamification elements for varying anticipated commitment |
| Jacobs, 2013 [12] | Gamification: A framework for the workplace |
| Julius, 2013 [13] | Designing gamification |
| Kumar, 2013 [14] | A framework for designing gamification in the enterprise |
| **Kumar, 2013** [15] | Gamification at Work: Designing Engaging Business Software |
| **Marache-Francisco and Brangier, 2013** [16] | Process of Gamification. From the Consideration of Gamification to its Practical Implementation |
| Gears D and Braun K, 2013 [17] | Gamification in business: Designing motivating solutions to problem situations |
| Chou YK, 2013 [18] | Octalysis: Complete gamification framework |
| Jiménez, 2013 [19] | Gamification model canvas |
| DiTommaso and Taylor, 2014 [20] | Beyond Gamification: Designing Behavior Change Games |
| Li, 2014 [21] | Evaluation of a theoretical model for gamification in workplace IS context |
| Burke, 2014 [22] | Gamify: how gamification motivates people to do extraordinary things |
| Herger, 2014 [23] | Enterprise Gamification: Engaging People by Letting Them Have Fun |
| Fitz-Walter, 2015 [24] | Achievement unlocked: Investigating the design of effective gamification experiences for mobile applications and devices |
| Helms, Barneveld and Dalpiaz, 2015 [25] | A Method for the Design of Gamified Trainings |
| Herzig, Ameling, Wolf and Schill, 2015 [26] | Implementing Gamification: Requirements and Gamification Platforms |
| AlMarshedi, Wanick, Wills and Ranchhod, 2015 [27] | SGI: a framework for increasing the sustainability of gamification impact |
| **Robson, Plangger, Kietzmann, McCarthy and Pitt, 2015** [28] | Is it all a game? Understanding the principles of gamification |
| **Brito, Vieira and Duran, 2015** [29] | Towards a Framework for Gamification Design on Crowdsourcing Systems: The G.A.M.E. Approach |
| **Deterding, 2015** [30] | The lens of intrinsic skill atoms: a method for gameful design |
| **Klevers, Sailer and Günthner, 2016** [31] | Implementation Model for the Gamification of Business Processes: A Study from the Field of Material Handling |
| Schmidt, Brosius and Herrmanny, 2017 [32] | Ein Vorgehensmodell für angewandte Spielformen |
| **Li, 2018** [33] | A Method to Support Gamification Design Practice with Motivation Analysis and Goal Modeling |
| **Liu D, Santhanam and Webster, 2018** [34] | Toward meaningful engagement: a framework for design and research of gamified information systems |
| **Morschheuser, Hassan, Werder and Hamari, 2018** [35] | How to design gamification? A method for engineering gamified software |

**Supplementary Table 2. The obtained common Pattern for gamifying by synthesizing 9 out of 11 selected gamification design frameworks**

| **Frameworks** | **Steps** | | | | | | | | | | |
| --- | --- | --- | --- | --- | --- | --- | --- | --- | --- | --- | --- |
|  | Step 1 | Step 2 | | Step 3 | | | | | | Step 4 | Step 5 |
|  | Objective determination | Knowing the users (1) and context of gamification (2) | | Generation of Concept/idea | | Game mechanics selection | | Prototyping | | Implementation | Monitoring |
|  |  | 1 | 2 | Iterative | Non iterative | Iterative | Non iterative | Iterative | Non iterative |  |  |
| Aparicio et al, 2012 [6] | √ | √ | √ | × | × | × | √ | × | × | √ | × |
| Marache-Francisco and Brangier, 2013 [16] | √ | √ | √ | √ | × | √ | × | √ | × | √ | × |
| Deterding, 2015 [30] | √ | √ | √ | √ | × | × | √ | √ | × | √ | × |
| Brito, Vieira and Duran, 2015 [29] | √ | √ | √ | √ | × | √ | × | × | × | √ | × |
| Kumar, 2013 [15] | √ | √ | √ | × | × | × | √ | × | × | × | √ |
| Morschheuser et al., 2018 [35] | √ | √ | √ | √ | × | √ | × | √ | × | √ | √ |
| Li, 2018 [33] | √ | √ | √ | × | × | × | √ | × | × | × | × |
| Werbach and  Hunter, 2012 [7] | √ | √ | √ | × | × | √ | × | √ | × | √ | × |
| Liu, Santhanam, and  Webster, 2017 [34] | √ | √ | √ | × | × | × | √ | × | × | × | × |

**References**

1. Hunicke R, LeBlanc M, Zubek R. MDA: A formal approach to game design and game research. In: Proceedings of the AAAI Workshop on Challenges in Game AI 2004; San Jose, CA. [[Google Scholar](https://scholar.google.com/scholar?hl=en&as_sdt=0%2C5&q=MDA%3A+A+formal+approach+to+game+design+and+game+research&btnG=)]
2. Charles D, Black M. Dynamic player modeling: A framework for player-centered digital games. In: Proceeding of the International Conference on Computer Games: Artificial Intelligence, Design and Education 2004; p. 29-35. [[Google Scholar](https://scholar.google.com/scholar?hl=en&as_sdt=0%2C5&q=Dynamic+player+modeling%3A+A+framework+for+player-centered+digital+games&btnG=)]
3. Dignan A. Game frame: Using games as a strategy for success. New York, NY: Free Press; 2011. [[Google Scholar](https://scholar.google.com/scholar?hl=en&as_sdt=0%2C5&q=Game+frame%3A+Using+games+as+a+strategy+for+success&btnG=)]
4. Radoff J. Game On: Energize Your Business with Social Media Games. Hoboken, New Jersey: Wiley, John & Sons, Incorporated; 2011:1-432. ISBN: 0470936266
5. Kapp KM. The gamification of learning and instruction: game-based methods and strategies for training and education. John Wiley & Sons; 2012. ISBN: 1118096347
6. Aparicio AF, Vela FLG, Sánchez JLG, Montes JLI. Analysis and application of gamification. In: Proceedings of the 13th International Conference on Interacción Persona-Ordenador. 2012 Presented at: Interacción 2012; October 03-05, 2012; Elche, Spain. [[CrossRef](https://dx.doi.org/10.1145/2379636.2379653)]
7. Werbach K, Hunter D. For The Win: How Game Thinking Can Revolutionize Your Business. Pennsylvania, USA: Wharton Digital Press; 2012:1-148. ISBN: 9781613630228
8. Versteeg C. Ethics & Gamification design a moral framework for taking responsibility. Utrech University, Utrecht; 2013. [[Google Scholar](https://scholar.google.com/scholar?hl=en&as_sdt=0%2C5&q=+Ethics+%26+Gamification+design+a+moral+framework+for+taking+responsibilit&btnG=)]
9. Marczewski A. Gamification: a simple introduction. Andrzej Marczewski, 2013; [[Google Scholar](https://scholar.google.com/scholar?hl=en&as_sdt=0%2C5&q=Gamification%3A+a+simple+introduction&btnG=)] ISBN: 1471798666
10. Merino de Paz B. Gamification: A tool to improve sustainability efforts. University of Manchester; 2013. [[Google Scholar](https://scholar.google.com/scholar?hl=en&as_sdt=0%2C5&q=Gamification%3A+A+tool+to+improve+sustainability+efforts&btnG=)]
11. Robinson D, Bellotti V. A preliminary taxonomy of gamification elements for varying anticipated commitment. In: Proceedings of the CHI 2013 Workshop on Designing Gamification: Creating Gameful and Playful Experiences 2013. [[Google Scholar](https://scholar.google.com/scholar?cluster=5431978241833471865&hl=en&as_sdt=0,5&sciodt=0,5)]
12. Jacobs H. Gamification: A framework for the workplace. University of Liverpool, Liverpool; 2013. [[Google Scholar](https://scholar.google.com/scholar?hl=en&as_sdt=0%2C5&q=Gamification%3A+A+framework+for+the+workplace&btnG=)]
13. Julius K. Designing gamification. University of Oulu, Business school marketing; 2013. [[Google Scholar](https://scholar.google.com/scholar?cluster=3514869543893696864&hl=en&as_sdt=0,5&sciodt=0,5)]
14. Kumar N. A framework for designing gamification in the enterprise. Infosys Labs Briefings 2013;11(3):8-13. [[Google Scholar](https://scholar.google.com/scholar?hl=en&as_sdt=0%2C5&q=.+A+framework+for+designing+gamification+in+the+enterprise&btnG=)]
15. Kumar J. Gamification at work: Designing engaging business software. In: international conference of design, user experience, and usability 2013; Springer, Berlin, Heidelberg p. 528-537. [[CrossRef](https://dx.doi.org/10.1007/978-3-642-39241-2_58)]
16. Marache-Francisco C, Brangier E. Process of Gamification. From the Consideration of Gamification to its Practical Implementation. In: CENTRIC 2013, Sixth International Conference on Advances in Human-oriented and Personalized Mechanisms, Technologies, and Services 2013; Venice, Italy p. 527-537.
17. Gears D, Braun K. Gamification in business: Designing motivating solutions to problem situations. In: Proceedings of the CHI 2013 Gamification Workshop 2013. [[Google Scholar](https://scholar.google.com/scholar?hl=en&as_sdt=0%2C5&q=Gamification+in+business%3A+Designing+motivating+solutions+to+problem+situations&btnG=)]
18. Chou YK. Octalysis: complete gamification framework. Gamification & Behavioral Design. URL: <http://yukaichou.com/gamification-examples/octalysis-complete-gamification-framework/>. [accessed 2020-09-17]
19. Jiménez S. Gamification model canvas. URL: <https://www.gamasutra.com/blogs/SergioJimenez/20131106/204134/Gamification_Model_Canvas.php> [accessed 2019-11-30]
20. DiTommaso D, Taylor C. Beyond Gamification: Designing Behavior Change Games. In: Proceedings of the first ACM SIGCHI annual symposium on Computer-human interaction in play 2014; Toronto, Ontario, Canada p. 475–475. [[CrossRef](https://dx.doi.org/10.1145/2658537.2662410)]
21. Li, C. Evaluation of a theoretical model for gamification in workplace IS context. University of British Columbia, Vancouver; 2014. [[Google Scholar](https://scholar.google.com/scholar?hl=en&as_sdt=0%2C5&q=Evaluation+of+a+theoretical+model+for+gamification+in+workplace+IS+context.+University+of+British+Columbia&btnG=)]
22. Burke B. Gamify: how gamification motivates people to do extraordinary things. Brookline, MA: Bibliomotion; 2014.
23. Herger M. Enterprise Gamification: Engaging People by Letting Them Have Fun. CreateSpace Independent Publishing Platform, Leipzig; 2014. [[Google Scholar](https://scholar.google.com/scholar?hl=en&as_sdt=0%2C5&q=+Enterprise+Gamification%3A+Engaging+people+by+letting+them+have+fun%2C+CreateSpace&btnG=)] ISBN: 1470000644
24. Fitz-Walter ZJ. Achievement unlocked: Investigating the design of effective gamification experiences for mobile applications and devices. Queensland University of Technology; 2015. [[Google Scholar](https://scholar.google.com/scholar?hl=en&as_sdt=0%2C5&q=Achievement+Unlocked%3A+Investigating+the+Design+of+Effective+Gamification+Experiences+for+Mobile+Applications+and+Devices&btnG=)]
25. Helms RW, Barneveld R, Dalpiaz F. A Method for the Design of Gamified Trainings. In: Proceedings of the PACIS 2015; Singapore, AIS. [[Google Scholar](https://scholar.google.com/scholar?hl=en&as_sdt=0%2C5&q=A+method+for+the+design+of+gamified+trainings&btnG=)]
26. Herzig P, Ameling M, Wolf B, Schill A. Implementing Gamification: Requirements and Gamification Platforms. In: Reiners T, Wood L (eds) Gamification in Education and Business; Springer, Cham p. 179-204. [[CrossRef](https://dx.doi.org/10.1007/978-3-319-10208-5_22)]
27. AlMarshedi A, Wanick V, Wills G, Ranchhod A. SGI: a framework for increasing the sustainability of gamification impact. Int J Infonomics 2015;8:1044–1052. [[Google Scholar](https://scholar.google.com/scholar?hl=en&as_sdt=0%2C5&q=SGI%3A+a+framework+for+increasing+the+sustainability+of+gamification+impact&btnG=)]
28. Robson K, Plangger K, Kietzmann JH, McCarthy I, Pitt L. Is it all a game? Understanding the principles of gamification. Bus Horiz 2011;58(4):411–420. [[CrossRef](https://dx.doi.org/10.1016/j.bushor.2015.03.006)]
29. Brito J, Vieira V, Duran A. Towards a Framework for Gamification Design on Crowdsourcing Systems: The G.A.M.E. Approach. In: Proceeding of 12th International Conference on Information Technology - New Generations 2015; Las Vegas, NV, USA. [[CrossRef](https://dx.doi.org/10.1109/ITNG.2015.78)]
30. Deterding S. The lens of intrinsic skill atoms: a method for gameful design. Hum-Comput Interact 2015 May 15;30(3-4):294-335. [[CrossRef](https://dx.doi.org/10.1080/07370024.2014.993471)]
31. Klevers M, Sailer M, Günthner WA. Implementation Model for the Gamification of Business Processes: A Study from the Field of Material Handling. In: Kaneda T, Kanegae H, Toyoda Y, Rizzi P (eds) Simulation and Gaming in the Network Society, Translational Systems Sciences 2016; vol 9. Springer, Singapore. [[Google Scholar](https://scholar.google.com/scholar?hl=en&as_sdt=2005&sciodt=0%2C5&cites=15831954303053138032&scipsc=&q="Implementation+Model+for+the+Gamification+of+Business+Processes%3A+A+Study+from+the+Field+of+Material+Handling"+and+jmir&btnG=)]
32. Schmidt R, Brosius C, Herrmanny K. Ein Vorgehensmodell für angewandte Spielformen. In: Gamification und Serious Games 2017; 52 (6): 826–839. [[CrossRef](https://dx.doi.org/10.1365/s40702-015-0180-y)]
33. Li X. A Method to Support Gamification Design Practice with Motivation Analysis and Goal Modeling. In: proceedings of the 2nd International GamiFIN Conference 2018; Pori, Finland p. 151–158.
34. Liu D, Santhanam R, Webster J. Toward meaningful engagement: a framework for design and research of gamified information systems. MIS Q 2017 Apr 4;41(4):1011-1034.
35. Morschheuser B, Hassan L, Werder K, Hamari J. How to design gamification? A method for engineering gamified software. Inf Softw Technol 2018;95:219–237. [[CrossRef](https://dx.doi.org/10.1016/j.infsof.2017.10.015)]
