# Supplementary material for: A Gamification Framework for Cognitive Assessment and Cognitive Training: Qualitative Study
Source: JMIR Serious Games. 2021 May 18;9(2):e21900. doi: 10.2196/21900 (PMC8170558; doi:10.2196/21900)
Supplement: Multimedia Appendix 2 [file games_v9i2e21900_app2.doc]

**Multimedia Appendix 2**

**Supplementary Table 1. List of final included papers applied gamification in cognitive training/ testing, the main issues of them are summarized a**

| **Study** | **Game up/ mapping/ making** | **Empirical project report/ theoretical** | **Testing/ training** | **Levels or Difficulty level** | **Comparing**  **to standard tasks or non gamified versions** | **Mini games** | **Technology** |
| --- | --- | --- | --- | --- | --- | --- | --- |
| Boendermaker, Prins and Wiers, 2015 [1] | Game up, mapping | Theoretical | Training | × | √ | × | × |
| Boendermaker, Boffo and Wiers, 2015 [2] | Game up | Empirical | Training | √ | √ | × | Smartphone |
| Lumsden et al, 2016 [3] | Game up | Theoretical | Both | √ | √ | √ | √ |
| Lumsden et al, 2016 [4] | Game up | Empirical | Testing | × | √ | × | × |
| Lumsden et al, 2017 [5] | Game up | Empirical | Testing | × | √ | × | × |
| Miranda and Palmer, 2013 [6] | Game up | Empirical | Training | × | √ | × | × |
| Katz et al, 2014 [7] | Game up | Empirical | Training | √ | √ | × | × |
| Hawkins et al, 2013 [8] | Game up | Empirical | Testing | √ | √ | × | × |
| Ninaus et al, 2015 [9] | Game up | Empirical | Training | √ | √ | × | × |
| Birk et al, 2015 [10] | Game up | Empirical | Testing | × | √ | √ | × |
| Tong and Chignell, 2014 [11] | Mapping | Empirical | Testing | √ | √ | × | Tablet |
| Tong, Chignell and Sieminowski, 2015 [12] | Mapping | Empirical | Testing | √ | √ | × | Tablet |
| Tong et al, 2016 [13] | Mapping | Empirical | Testing | √ | √ | × | Tablet |
| Tong, Chan and Chignell, 2017 [14] | Game up, mapping | Theoretical / empirical | Testing | × | √ | × | Tablet |
| Valladares-Rodriguez et al, 2016 [15] | Game up, mapping | Theoretical | Testing | √ | √ | × | VR (virtual reality), touch devices |
| Valladares-Rodriguez et al, 2017 [16] | Game up | Empirical | Testing | √ | √ | × | VR, touch devices |
| Valladares-Rodriguez et al, 2018 [17] | Game up, mapping | Empirical | Testing | √ | √ | √ | VR, touch devices |
| Craven and Groom, 2015 [18] | Game up | Theoretical prototype | Both | √ | × | × | × |
| Boot, 2015 [19] | Mapping | Theoretical | Training | √ | × | × | × |
| Green and Bavelier, 2012 [20] | Game up, mapping | Theoretical | Training | √ | × | √ | × |
| Gielis et al, 2017 [21] | Mapping | Empirical | Testing | √ | × | × | × |
| Jimison et al, 2004 [22] | Mapping | Empirical | Testing | √ | × | × | × |
| Bavelier et al, 2011 [23] | Mapping | Theoretical | Testing | √ | × | × | × |
| Shaban and Pearson, 2019 [24] | Game up | Theoretical framework | Training | √ | × | √ | √ |
| Zeng et al, 2018 [25] | Game up | Empirical | Testing | × | × | √ | × |
| Bavelier, Green and Seidenberg, 2013 [26] | Mapping | Theoretical | Training | × | × | × | × |
| Tenorio Delgado et al, 2014 [27] | Game up | Empirical | Testing | × | √ | √ | Touch screen devices |
| Joselli et al, 2014 [28] | Mapping | Empirical | Training | √ | × | × | EEG, smart phone |
| Joselli et al, 2017 [29] | Mapping | Empirical | Training | √ | × | × | EEG, smart phone |
| Dörrenbächer et al, 2014 [30] | Making | Empirical | Training | √ | √ | × | × |
| Dörrenbächer and Kray, 2018 [31] | Making | Empirical | Training | √ | √ | × | × |
| Prins et al, 2011 [32] | Game up | Empirical | Training | √ | √ | × | × |
| Prins et al, 2013 [33] | Game up | Empirical | Training | √ | × | √ | × |
| McPHERSON and BURNS, 2007 [34] | Game up | Empirical | Training | × | √ | × | × |
| McPHERSON and BURNS, 2008 [35] | Game up | Empirical | Training | × | √ | × | × |
| Baniqued et al, 2012 [36] | Game up, mapping | Empirical | Training | √ | √ | √ | × |
| Doherty et al, 2018 [37] | Mapping | Theoretical | Training | × | × | × | × |
| Bavelier and Green, 2016 [38] | Mapping | Theoretical | Training | √ | √ | × | × |
| Gamberini et al, 2009 [39] | Mapping | Empirical | Testing | √ | √ | √ | Tangible interface (tabletop) |
| Robert et al, 2014 [40] | × | Theoretical | Both | √ | × | × | VR |
| Vourvopoulos et al, 2014 [41] | Game up | Empirical | Both | √ | √ | × | VR |
| Vasiljevic and de Miranda, 2019 [42] | Mapping | Theoretical | Training | √ | × | √ | Brain computer interface (BCI) |
| Dye, Green, and Bavelier, 2009 [43] | Mapping | Theoretical | Training | × | × | × | × |
| Cota, Ishitani, and Vieira Jr, 2015 [44] | Making | Empirical | Training | √ | × | √ | Smart phone |
| Groznik and Sadikov, 2019 [45] | Game up, mapping | Theoretical | Both | √ | √ | × | VR |
| Söderberg, 2011 [46] | Game up, mapping | Empirical | Training | √ | × | √ | √ |
| Mandryk, 2012 [47] | Mapping | Empirical | Training | √ | × | × | Smart phone,  EEG |
| Goh, Ang and Tan, 2008 [48] | × | Theoretical | Training | √ | × | × | VR |
| Manera et al, 2015 [49] | Making | Empirical | Both | × | √ | √ | Tablet |
| Green and Bavelier, 2015 [50] | Mapping | Theoretical | Training | √ | √ | × | × |
| Franceschini et al, 2013 [51] | Mapping | Empirical | Training | × | √ | √ | × |
| Green and Bavelier, 2003 [52] | Mapping | Empirical | Training | √ | √ | × | × |
| Verhaegh et al, 2012 [53] | Game up | Empirical | Both | √ | √ | × | Tangible interface (Tagtile technology) |
| Aarts et al, 2019 [54] | Mapping | Empirical | Both | √ | √ | × | Touch sensitive devices |
| Jaramillo-Alcázar, Luján-Mora and Salvador-Ullauri, 2018 [55] | × | Theoretical | Implicitly related to the both | √ | × | × | √ |
| Tarnanas et al, 2015 [56] | Game up | Empirical | Testing | √ | × | √ | VR |
| Peijnenborgh et al, 2016 [57] | Game up | Empirical | Testing | √ | × | √ | × |
| Aalbers et al, 2013 [58] | Game up | Empirical | Testing | √ | √ | √ | × |
| Brown et al, 2014 [59] | Game up | Empirical | Testing | √ | × | √ | Smart phone |
| Anguera and Gazzaley, 2015 [60] | Game up, mapping | Theoretical | Training | √ | √ | √ | × |
| Van de Weijer-Bergsma et al, 2014 [61] | Game up | Empirical | Testing | √ | √ | × | × |
| Brilliant T, Nouchi and Kawashima, [62] | Mapping | Theoretical | Training | × | × | × | × |
| Navan and Khaleghi, 2020 [63] | Game up | Empirical | Training | × | × | × | × |

^a^ Technology coulumn indicates whether or not the articles have paid attention to select appropriate technologies or use interfaces and devices that allow their users natural interaction.

**Supplementary Table 2.** Studies have investigated gamification in cognitive contexts are categorized by target cognitive area and population

| **Study** | **Target cognitive area and population** |
| --- | --- |
| Boendermaker, Prins and Wiers, 2015 [1] | Cognitive bias modification, adolescents |
| Boendermaker, Boffo and Wiers, 2015 [2] | Cognitive bias modification, young adults  (A sample of regularly drinking undergraduate students  (n=77; mean – standard deviation [SD] age, 22.7–3.1 years; age range, 18–29 years, 50.6 percent male)) |
| Lumsden et al, 2016 [3] | Focuses on application and efficacy of gamified cognitive assessment and training paradigms |
| Lumsden et al, 2016 [4] | Response inhibition, 84 subjects in the laboratory (mean age = 21, standard deviation (SD)= 4, 26% male) and 203 subjects online (mean age = 35, SD = 11, 50% male) |
| Lumsden et al, 2017[5] | Response inhibition, experiment 1: 91 subjects,  experiment 2 : 86 subjects, experiment 3: 83 subjects (older than 18 years) |
| Miranda and Palmer, 2013 [6] | Attentional capture ability, experiment 1: 24 subjects (mean age= 21.55, SD = 4.37), experiment 2: 31 subjects (mean age = 22.55, SD = 5.76), experiment 3: 22 subjects (mean= 20.95, SD = 2.95) |
| Katz et al, 2014 [7] | Working memory, 107 school-based students (mean age = 10.65 years, SD = 2.36, range 6–14, 44% girls) |
| Hawkins et al, 2013 [8] | experiment 1: targeting choice cognitive task, 200 first-year psychology students  experiment 2: targeting detection cognitive task, 100 first-year psychology students |
| Ninaus et al, 2015 [9] | Working memory, 39 university students (mean age = 23.8 years, 7 male, 32 female, SD = 5.05) |
| Birk et al, 2015 [10] | Executive functioning, short-term memory, emotion recognition, and identifying ambiguous word,  218 subjects ( mean: 32.79 , SD: 10.29, 46.3% female) |
| Tong and Chignell, 2014 [11] | Assessing abilities on central executive functions within working memory, adults  (7 females and 17 males between the ages of 21 to 51 years) |
| Tong, Chignell and Sieminowski, 2015 [12] | Patients with cognitive deficits  (21participants aged over 18) |
| Tong et al, 2016 [13] | Examined the feasibility of a game-based cognitive assessment presented on tablet technology against standard mental status tools, elderly adults (N=146; age: mean 80.59, SD 6.00, range 70-94 years) |
| Tong, Chan and Chignell, 2017 [14] | Dementia, aging populations |
| Valladares-Rodriguez et al, 2016 [15] | Investigated on efficacy of serious games for cognitive assessment |
| Valladares-Rodriguez et al, 2017 [16] | Episodic memory, senior adults  (eight people without cognitive impairments or healthy control group (average age of 68.3 ± 8.88 years); (2) five AD patients (average age of 75.8 ± 5.36 years); and finally, three MCI patients (average age of 75 ± 6.08 years)) |
| Valladares-Rodriguez et al, 2018 [17] | AD and MCI, older adults (average age 68.3±8.88 years); (2) 5 AD patients (aver- age age 75.8±5.36 years, and (3), 3 MCI patients (average age 75±6.08 years) |
| Craven and Groom, 2015 [18] | ADHD, children |
| Boot, 2015 [19] | Focuses on the impacts of video games on cognitive processes |
| Green and Bavelier, 2012 [20] | Focuses on the effects of action video games on learning new tasks, attentional control abilities and etc. |
| Gielis et al, 2017 [21] | Mild cognitive impairment, older adults |
| Jimison et al, 2004 [22] | Detecting cognitive status in elders (mean age of 80.2 8.0 +/- 8.0) |
| Bavelier et al, 2011 [23] | Focuses on the negative and positive effects of video games |
| Shaban and Pearson, 2019 [24] | Learning disabilities, children |
| Zheng et al, 2018 [25] | Dementia, seniors  (5 healthy older adults aged above 60) |
| Bavelier, Green and Seidenberg, 2013 [26] | Italian dyslexic children (N = 20) |
| Tenorio Delgado et al, 2014 [27] | Assessing cognitive abilities, children (Fifty-four (54) children between 3 and 9 years of age) |
| Joselli et al, 2014 [28] | Attention ability, 11 subjects (6 to 42 years with and without cognitive disorders) |
| Joselli et al, 2017 [29] | Attention ability, 11 subjects (6 to 42 years with and without cognitive disorders) |
| Dörrenbächer et al, 2014 [30] | Task-switching abilities, middle childhood |
| Dörrenbächer and Kray, 2018 [31] | ADHD, children (54 children (age range = 8.10–11.10 sample was subdivided into four different training groups which years, mean age = 9.64 years, SD = 0.80, 48.1% female) |
| Prins et al, 2011 [32] | ADHD, children (6 and 12 years) |
| Prins et al, 2013 [33] | ADHD, children (8–12 years) |
| McPHERSON and BURNS, 2007 [34] | Processing speed (Gs) and working memory (WM),  experiment 1: 70 Level I psychology students (mean: 19.6 years, SD: 4.00), experiment 2: 94 children 11 to 14 years (mean:12.88, SD: 0.80) |
| McPHERSON and BURNS, 2008 [35] | Processing speed, experiment 1: 60 Level I psychology students (mean: 20.6 years, SD: 2.81), experiment 2: 61 Level I Psychology students (mean: 20.0 years, SD: 3.56) |
| Baniqued et al, 2012 [36] | Cognitive abilities,  Ages (18-30) |
| Doherty et al, 2018 [37] | The impacts of video games on cognition abilities |
| Bavelier and Green, 2016 [38] | Focuses on the powers of video games on improving cognitive abilities |
| Gamberini et al, 2009 [39] | Preserving cognitive functions in elderly people  (Mean Age = 68.083, Range = 64-75, Standard Deviation = 3.34) |
| Robert et al, 2014 [40] | Elderly people and people with dementia-related disorders |
| Vourvopoulos et al, 2014 [41] | People with cognitive deficits  (10 patients (8 females and 2 males between 35 and 77 years old) with cognitive deficits derived from stroke, traumatic brain injury and mild cognitive impairment) |
| Vasiljevic and de Miranda, 2019 [42] | Examined brain interface games based on consumer-grade EEG devices |
| Dye, Green, and Bavelier, 2009 [43] | Processing speed,  young adults |
| Cota, Ishitani, and Vieira, 2015 [44] | Elderly (60- 69) |
| Groznik and Sadikov, 2019 [45] | Mild cognitive impairment, older adults |
| Söderberg, 2011 [46] | Cognitive impaired children (6 persons (10-12 years) |
| Mandryk, 2012 [47] | Using biofeedback games for training fetal alcohol spectrum disorder and ADHD patients |
| Goh, Ang and Tan, 2008 [48] | Mental health problems,  child and adolescents |
| Manera et al, 2015 [49] | Mild cognitive impairment (MCI) and alzheimer’s disease, elderly people (Participants Nine MCI patients (seven female and two male; mean age = 75.8 years; SD = 9.1; age range = 60–84) and 12 patients with AD or related disorders (eight female, four male; mean age = 80.3 years; SD = 6.3; age range = 70–90) voluntarily) |
| Green and Bavelier, 2015 [50] | Using action video games training for improving cognitive abilities |
| Franceschini et al, 2013 [51] | Dyslexia, children |
| Green and Bavelier, 2003 [52] | Using action video games for modifying visual selective attention ability |
| Verhaegh et al, 2012 [53] | Nonverbal cognitive skills, children  (children aged 8–10 years and consisted of a pilot study (N = 10) and an experiment (N=32)) |
| Aarts et al, 2019 [54] | Visual search ability, youth, adults, senior  (28 healthy younger (20 female and 8 male) aged between 18 and 31 years (mean 21.68 years, SD 2.86), 13 healthy older adults (7 female and 6 male) aged between 64 and 79 years (mean 70.54 years, SD 3.82), and 11 oldest adults (9 female and 2 male) aged between 86 and 94 years (mean 89.27, SD 3.29)) |
| Jaramillo-Alcázar, Luján-Mora and Salvador-Ullauri, 2018 [55] | People with cognitive disabilities |
| Tarnanas et al, 2015 [56] | Patient with amnestic mild cognitive impairment (aMCI)  (25 aMCI (18 females and 7 males) and 25 con trols (20 females and 5 males) with a mean age of 64.3 years) |
| Peijnenborgh et al, 2016 [57] | ADHD , children (4-8 years) |
| Aalbers et al, 2013 [58] | Working memory, visuospatial short-term memory, episodic recognition memory, aging adults |
| Brown et al, 2014 [59] | Working memory , attentional blink, inhibitory and decision-making |
| Anguera and Gazzaley, 2015 [60] | Cognitive abilities |
| Van de Weijer-Bergsma et al, 2014 [61] | visual– spatial working memory, updating, shifting and inhibition abilities  school-aged children (6–12 years of age) |
| Brilliant T, Nouchi and Kawashima, 2019 [62] | The impact of video games on brain |
| Navan and Khaleghi, 2020 [63] | Training emotional states recognition of autistic children  10 children (6-14 years of age) |

**References**

1.Boendermaker WJ, Prins PJM, Wiers RW. Cognitive Bias Modification for adolescents with substance use problems - Can serious games help? J Behav Ther Exp Psychiatry 2015;49 (Pt A):13–20. [[CrossRef](https://dx.doi.org/10.1016/j.jbtep.2015.03.008)] [[Medline](https://www.ncbi.nlm.nih.gov/pubmed/?term=Cognitive+Bias+Modification+for+adolescents+with+substance+use+problems+-+Can+serious+games+help%3F)]

2. Boendermaker WJ, Boffo M, Wiers RW. Exploring Elements of Fun to Motivate Youth to Do Cognitive Bias Modification. Games Health J 2015;4(6):434–443. [[CrossRef](https://dx.doi.org/10.1089/g4h.2015.0053)][[Medline](https://www.ncbi.nlm.nih.gov/pubmed/?term=Exploring+Elements+of+Fun+to+Motivate+Youth+to+Do+Cognitive+Bias+Modification)]

3. Lumsden J, Edwards EA, Lawrence NS, Coyle D, Munafò MR. Gamification of cognitive assessment and cognitive training: a systematic review of applications and efficacy. JMIR serious games 2016;4(2):e11. [[CrossRef](https://dx.doi.org/ 10.2196/games.5888)] [[Medline](https://www.ncbi.nlm.nih.gov/pubmed/?term=Gamification+of+Cognitive+Assessment+and+Cognitive+Training%3A+A+Systematic+Review+of+Applications+and+Efficacy)]

4. Lumsden J, Skinner A, Woods AT, Lawrence NS, Munafò M. The effects of gamelike features and test location on cognitive test performance and participant enjoyment. PeerJ 2016;4:e2184. [doi: [10.7717/peerj.2184](https://peerj.com/articles/2184/)] [Medline: [27441120](https://www.ncbi.nlm.nih.gov/pubmed/?term=The+effects+of+gamelike+features+and+test+location+on+cognitive+test+performance+and+participant+enjoyment)]

5. Lumsden J, Skinner A, Coyle D, Lawrence N, Munafo M. Attrition from Web-Based Cognitive Testing: A Repeated Measures Comparison of Gamification Techniques. J Med Internet Res 2017;19(11):e395. [[CrossRef](https://dx.doi.org/ 10.2196/jmir.8473)][[Medline](https://www.ncbi.nlm.nih.gov/pubmed/?term=Attrition+from+Web-Based+Cognitive+Testing%3A+A+Repeated+Measures+Comparison+of+Gamification+Techniques)]

6. Miranda AT, Palmer EM. Intrinsic motivation and attentional capture from gamelike features in a visual search task. Behav Res Methods 2013;46(1):159–172. [[CrossRef](https://dx.doi.org/10.3758/s13428-013-0357-7)][[Medline](https://www.ncbi.nlm.nih.gov/pubmed/?term=Intrinsic+motivation+and+attentional+capture+from+gamelike+features+in+a+visual+search+task)]

7. Katz B, Jaeggi S, Buschkuehl M, Stegman A, Shah P. Differential effect of motivational features on training improvements in school-based cognitive training. Front Hum Neurosci 2014;8: 242. [[CrossRef](https://dx.doi.org/10.3389/fnhum.2014.00242) ][[Medline](https://www.ncbi.nlm.nih.gov/pubmed/?term=Differential+effect+of+motivational+features+on+training+improvements+in+school-based+cognitive+training)]

8. Hawkins GE, Rae B, Nesbitt K V, Brown SD. Gamelike features might not improve data. Behav Res Methods 2013;45(2):301–318. [[CrossRef](https://dx.doi.org/10.3758/s13428-012-0264-3)]

9. Ninaus M, Pereira G, Stefitz R, Prada R, Paiva A, Neuper C, et al. Game elements improve performance in a working memory training task. Int J Serious Games 2015;2(1):3–16. [[CrossRef](https://dx.doi.org/10.17083/ijsg.v2i1.60)]

10. Birk M V, Mandryk RL, Bowey J, Buttlar B. The effects of adding premise and Backstory to Psychological Tasks. In: CHI’15 Workshop Researching Gamification: Strategies, Opportunities, Challenges, Ethics 2015; Seoul. [[Google Scholar](https://scholar.google.com/scholar?hl=en&as_sdt=0%2C5&q=The+effects+of+adding+premise+and+Backstory+to+Psychological+Tasks&btnG=)]

11. Tong T, Chignell M. Developing a Serious Game for Cognitive Assessment: Choosing Settings and Measuring Performance. In: Proceedings of the Second International Symposium of Chinese CHI 2014 Apr 26; Toronto, Canada p. 70-79. [[CrossRef](https://dx.doi.org/10.1145/2592235.2592246)]

12. Tong T, Chignell M, Sieminowski T. Case study: a serious game for neurorehabilitation assessment. Procedia Computer Science 2015;69:125-131. [[CrossRef](https://dx.doi.org/10.1016/j.procs.2015.10.013)]

13. Tong T, Chignell M, Tierney MC, Lee J. A serious game for clinical assessment of cognitive status: validation study. JMIR Serious Games 2016;4(1):e7. [[CrossRef](https://dx.doi.org/10.2196/games.5006)] [[Medline](https://www.ncbi.nlm.nih.gov/pubmed/?term=A+Serious+Game+for+Clinical+Assessment+of+Cognitive+Status%3A+Validation+Study)]

14. Tong T, Chan JH, Chignell M. Serious Games for Dementia. In: proceedings of the 26th International Conference on World Wide Web Companion 2017; Perth, Australia p. 1111–1115. [[CrossRef](https://dx.doi.org/10.1145/3041021.3054930)]

15. Valladares-Rodríguez S, Pérez-Rodríguez R, Anido-Rifón L, Fernández-Iglesias M. Trends on the application of serious games to neuropsychological evaluation: A scoping review. J Biomed Inform 2016;64:296–319. [[CrossRef](https://dx.doi.org/10.1016/j.jbi.2016.10.019)] [[Medline](https://www.ncbi.nlm.nih.gov/pubmed/27815228)]

16. Valladares-Rodriguez S, Perez-Rodriguez R, Facal D, Fernandez-Iglesias MJ, Anido-Rifon L, Mouriño-Garcia M. Design process and preliminary psychometric study of a video game to detect cognitive impairment in senior adults. PeerJ 2017; 5:e3508. [[CrossRef](https://dx.doi.org/10.7717/peerj.3508)] [[Medline](https://www.ncbi.nlm.nih.gov/pubmed/?term=Design+process+and+preliminary+psychometric+study+of+a+video+game+to+detect+cognitive+impairment+in+senior+adults)]

17. Valladares-Rodriguez S, Pérez-Rodriguez R, Fernandez-Iglesias JM, Anido-Rifón LE, Facal D, Rivas-Costa C. Learning to Detect Cognitive Impairment through Digital Games and Machine Learning Techniques. Methods Inf Med 2018;57(04):197-207. [[CrossRef](https://dx.doi.org/10.3414/ME17-02-0011)]

18. Craven M, Groom M. Computer games for user engagement in Attention Deficit Hyperactivity Disorder (ADHD) monitoring and therapy. In: IEEE Computer Society Conference Proceedings. 2015 Oct Presented at: International Conference on Interactive Technologies and Games (iTAG); 22-23 October 2015; Nottingham, Nottinghamshire, United Kingdom p. 22-23. [[CrossRef](https://dx.doi.org/10.1109/iTAG.2015.9)]

19. Boot WR. Video games as tools to achieve insight into cognitive processes. Front Psychol 2015; 6: 3. [[CrossRef](https://dx.doi.org/10.3389/fpsyg.2015.00003)][[Medline](https://www.ncbi.nlm.nih.gov/pmc/articles/PMC4300862/)]

20. Green CS, Bavelier D. Learning, attentional control, and action video games. Curr Biol 2012;22(6):R197–R206. [[CrossRef](https://dx.doi.org/ 10.1016/j.cub.2012.02.012)][[Medline](https://www.ncbi.nlm.nih.gov/pubmed/22440805)]

21. Gielis K, Brito F, Tournoy J, Vanden Abeele V. Can Card Games Be Used to Assess Mild Cognitive Impairment? A Study of Klondike Solitaire and Cognitive Functions. In: CHI PLAY '17 Extended Abstracts Extended Abstracts Publication of the Annual Symposium on Computer-Human Interaction in Play 2017; Amsterdam, Netherlands p. 269-276. [[CrossRef](https://dx.doi.org/10.1145/3130859.3131328)]

22. Jimison H, Pavel M, McKanna J, Pavel J. Unobtrusive monitoring of computer interactions to detect cognitive status in elders. IEEE Trans Inf Technol Biomed 2004; 8(3):248-252. [[CrossRef](https://dx.doi.org/10.1109/titb.2004.835539)] [[Medline](https://www.ncbi.nlm.nih.gov/pubmed/15484429)]

23. Bavelier D, Green CS, Han DH, Renshaw PF, Merzenich MM, Gentile DA. Brains on video games. Nat Rev Neurosci 2011;12(12):763–768. [[CrossRef](https://dx.doi.org/10.1038/nrn3135)] [[Medline](https://www.ncbi.nlm.nih.gov/pubmed/22095065)]

24. Shaban A, Pearson E. A Learning Design Framework to Support Children with Learning Disabilities Incorporating Gamification Techniques. In: CHI EA '19 Extended Abstracts of the 2019 CHI Conference on Human Factors in Computing Systems 2019; Glasgow, Scotland UK. [[CrossRef](https://dx.doi.org/ 10.1145/3290607.3312806)]

25. Zeng Z, Fauvel S, Hsiang BTT, Wang D, Qiu Y, Khuan PCO, Leung C, Shen Z, Chin JJ. Towards long-term tracking and detection of early dementia: a computerized cognitive test battery with gamification. In: Proceedings of the 3rd International Conference on Crowd Science and Engineering 2018; Singapore, Singapore p. 28 - 31. [[CrossRef](https://dx.doi.org/10.1145/3265689.3265719)]

26. Bavelier D, Green CS, Seidenberg MS. Cognitive Development: Gaming Your Way Out of Dyslexia? Curr Biol 2013;23(7):R282–R283. [[CrossRef](https://dx.doi.org/10.1016/j.cub.2013.02.051)] [[Medline](https://www.ncbi.nlm.nih.gov/pubmed/?term=Cognitive+Development%3A+Gaming+Your+Way+Out+of+Dyslexia%3F)]

27. Delgado MT, Uribe PA, Alonso AA, Díaz RR. TENI: A comprehensive battery for cognitive assessment based on games and technology. Child Neuropsychol 2016;22(3):276-291. [[CrossRef](https://dx.doi.org/10.1080/09297049.2014.977241)] [[Medline](https://www.ncbi.nlm.nih.gov/pubmed/?term=TENI%3A+A+comprehensive+battery+for+cognitive+assessment+based+on+games+and+technology)]

28. Joselli M, Binder F, Clua E, Soluri E. MindNinja: Concept, development and evaluation of a mind action game based on EEGs. In: 2014 Brazilian Symposium on Computer Games and Digital Entertainment MindNinja 2014; Porto Alegre, Brazil p. 123-132. [[CrossRef](https://dx.doi.org/10.1109/SBGAMES.2014.14)]

29. Joselli M, Binder F, Clua E, Soluri E. Concept, development and evaluation of a mind action game with the electro encephalograms as an auxiliary input. SBC Journal on Interactive Systems 2017;8(1):60–73. [[Google Scholar](https://scholar.google.com/scholar?hl=en&as_sdt=0%2C5&q=Concept%2C+development+and+evaluation+of+a+mind+action+game+with+the+electro+encephalograms+as+an+auxiliary+input&btnG=)]

30. Dörrenbächer S, Müller PM, Tröger J, Kray J. Dissociable effects of game elements on motivation and cognition in a task-switching training in middle childhood. Front Psychol 2014;5:1275. [[CrossRef](https://dx.doi.org/10.3389/fpsyg.2014.01275)] [[Medline](https://www.ncbi.nlm.nih.gov/pubmed/?term=Dissociable+effects+of+game+elements+on+motivation+and+cognition+in+a+task-switching+training+in+middle+childhood)]

31. Dörrenbächer S, Kray J. The impact of game-based task-shifting training on motivation and executive control in children with ADHD. JCogn Enhanc 2018;3(1):64-84. [[CrossRef](https://dx.doi.org/10.1007/s41465-018-0083-2)]

32. Prins PJM, Dovis S, Ponsioen A, ten Brink E, van der Oord S. Does Computerized Working Memory Training with Game Elements Enhance Motivation and Training Efficacy in Children with ADHD? Cyberpsychol Behav Soc Netw 2011;14(3):115–122. [[CrossRef](https://dx.doi.org/10.1089/cyber.2009.0206)] [[Medline](https://www.ncbi.nlm.nih.gov/pubmed/20649448)]

33. Prins PJM, Brink E Ten, Dovis S, Ponsioen A, Geurts HM, de Vries M, et al. “Braingame Brian”: Toward an Executive Function Training Program with Game Elements for Children with ADHD and Cognitive Control Problems. Games Health J 2013;2(1):44–49. [[CrossRef](https://dx.doi.org/10.1089/g4h.2013.0004)] [[Medline](https://www.ncbi.nlm.nih.gov/pubmed/?term=“Braingame+Brian”%3A+Toward+an+Executive+Function+Training+Program+with+Game+Elements+for+Children+with+ADHD+and+Cognitive+Control+Problems)]

34. McPherson J, Burns NR. Gs Invaders: Assessing a computer game-like test of processing speed. Behav Res Methods 2007;39(4):876–883. [[CrossRef](https://dx.doi.org/10.3758/BF03192982)] [[Medline](https://www.ncbi.nlm.nih.gov/pubmed/?term=Assessing+the+validity+of+computer-game-like+tests+of+processing+speed+and+working+memory)]

35. McPherson J, Burns NR. Assessing the validity of computer-game-like tests of processing speed and working memory. Behav Res Methods 2008;40(4):969–981. [[CrossRef](https://dx.doi.org/ 10.3758/BRM.40.4.969)] [[Medline](https://www.ncbi.nlm.nih.gov/pubmed/?term=Assessing+the+validity+of+computer-game-like+tests+of+processing+speed+and+working+memory)]

36. Baniqued PL, Lee H, Voss MW, Basak C, Cosman JD, DeSouza S, et al. Selling points: What cognitive abilities are tapped by casual video games? Acta Psychol (Amst) 2013;142(1):74–86. [[CrossRef](https://dx.doi.org/ 10.1016/j.actpsy.2012.11.009)][[Medline](https://www.ncbi.nlm.nih.gov/pubmed/?term=Selling+points%3A+What+cognitive+abilities+are+tapped+by+casual+video+games%3F)]

37. Doherty SM, Keebler JR, Davidson SS, Palmer EM, Frederick CM. Recategorization of Video Game Genres. In: Proceedings of the Human Factors and Ergonomics Society Annual Meeting 2018; 62(1): 2099-2103. [[CrossRef](https://dx.doi.org/10.1177/1541931218621473)]

38. Bavelier D, Green CS. The Brain-Boosting Power of Video Games. Sci Am 2016; 315(1):26–31. [[CrossRef](https://dx.doi.org/10.1038/scientificamerican0716-26)] [[Medline](https://www.ncbi.nlm.nih.gov/pubmed/?term=The+Brain-Boosting+Power+of+Video+Games)]

39. Gamberini L, Martino F, Seraglia B, Spagnolli A, Fabregat M, Ibanez F, et al. Eldergames project: An innovative mixed reality table-top solution to preserve cognitive functions in elderly people. In: 2009 2nd Conference on Human System Interactions 2009; Catania, Italy p. 164-169. [[CrossRef](https://dx.doi.org/10.1109/HSI.2009.5090973)]

40. Robert PH, König A, Amieva H, Andrieu S, Bremond F, Bullock R, et al. Recommendations for the use of Serious Games in people with Alzheimer's Disease, related disorders and frailty. Front Aging Neurosci 2014 Mar 24;6:54. [[CrossRef](https://dx.doi.org/10.3389/fnagi.2014.00054)] [[Medline](https://www.ncbi.nlm.nih.gov/entrez/query.fcgi?cmd=Retrieve&db=PubMed&list_uids=24715864&dopt=Abstract)]

41. Vourvopoulos A, Faria AL, Ponnam K, Bermudez i Badia S. RehabCity: design and validation of a cognitive assessment and rehabilitation tool through gamified simulations of activities of daily living. In: Proceedings of the 11th conference on advances in computer entertainment technology 2014; New York, NY, USA. [[CrossRef](https://dx.doi.org/10.1145/2663806.2663852)]

42. Vasiljevic GAM, de Miranda LC. Brain–Computer Interface Games Based on Consumer-Grade EEG Devices: A Systematic Literature Review. Int J Hum Comput Interact 2019; 1–38. [[CrossRef](https://dx.doi.org/10.1080/10447318.2019.1612213)]

43. Dye MWG, Green CS, Bavelier D. Increasing Speed of Processing With Action Video Games. Curr Dir Psychol Sci 2009;18(6):321-326. [[CrossRef](https://dx.doi.org/10.1111/j.1467-8721.2009.01660.x)] [[Medline](https://www.ncbi.nlm.nih.gov/pubmed/20485453?dopt=Abstract)]

44.
